# Adrenal metastasis as first presentation of hepatocellular carcinoma

**DOI:** 10.1186/1477-7819-3-50

**Published:** 2005-07-25

**Authors:** Kostas Tsalis, Emmanouil Zacharakis, Nikolaos Sapidis, Ioannis Lambrou, Evangelos Zacharakis, Dimitrios Betsis

**Affiliations:** 1 4^th ^Surgical Department, Aristotle University of Thessaloniki, 'G. Papanikolaou' General Regional Hospital, Exohi, Thessaloniki 57010, Greece

## Abstract

**Background:**

Metastases from hepatocellular carcinoma (HCC) can be found in the lung and adrenal gland. We report case of a patient who presented with adrenal metastasis as the first clinical manifestation of HCC.

**Case presentation:**

A patient was referred for surgical treatment for a tumor in retro-peritoneal space. The computerized tomography (CT) scan revealed a mass originating from the left adrenal gland. The patient underwent left adrenalectomy and the exploration of abdominal cavity did not reveal any other palpable lesions. Histologically, the resected lesion was a poorly differentiated metastatic tumor from HCC. Seven months later patient was readmitted complaining of cachexia, icterus, and significant weight loss. CT scan revealed hyperdense lesions of the liver

**Conclusion:**

HCC may have atypical presentations like in present case. Fine needle aspiration/tru-cut^® ^biopsy might be useful in the investigation of an accidentally discovered adrenal mass regardless of the size and can lead to the detection of a primary tumor.

## Background

Metastases from hepatocellular carcinoma (HCC) can be found in the lung and adrenal gland [1]. It has been reported that adrenal metastases from HCC are found in 8.4 % of autopsy cases, being the second most common site of metastasis after the lung [1]. Recent advances in diagnostic techniques and therapeutic methods have contributed to an improved prognosis for patients with HCC [2].

Despite the fact that adrenal metastases from HCC are well documented, it is rare to have adrenal metastasis as the first clinical manifestation.

## Case presentation

A 76 year old man was refferd from a District Hospital for further treatment of a tumor in retro-peritoneal space, originating from the left adrenal gland.

**Figure 1:**
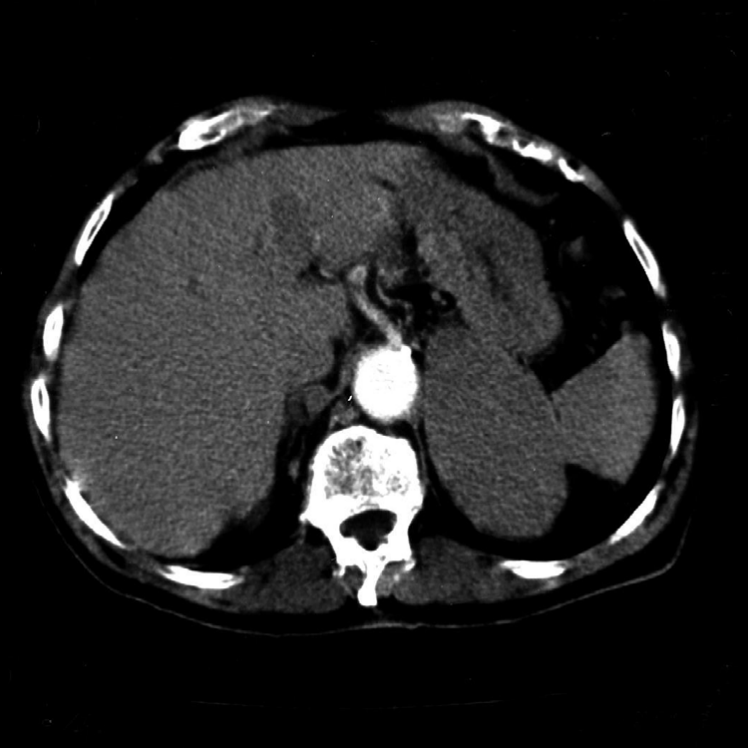
CT scan showing left adrenal gland tumor 10 cm in diameter, having homogenous intensity. No liver lesion is visualized.

The patient was admitted to the District Hospital for investigation of macroscopic haematuria and lumbar pain during the last 48 hours. He had a past medical history of hypertension and hepatitis B. The laboratory investigations revealed hypercalcaemia and in an abdominal ultrasound scan (US), a mass was detected in the retroperitoneal space close to the left kidney. Then a Computed Tomography (CT) scan with contrast was performed which revealed an enhancing mass, 10 cm in diameter, between the pancreas and the left kidney originating from the left adrenal gland. No other pathological findings from intra or extra-peritoneal organs were identified (Figure 1). The serum alpha-fetoprotein (AFP) concentration was 75.6 ng/ml (normal range: 0–10 ng/ml) and C19-9 concentration 37.6 ng/ml, while the liver function tests were normal.

After his admission to our Department, the patient was tested for underlying hormonally active tumor by low dose (1 mg) overnight dexamethasone suppression test and a 24-hour urine collection for catecholamines, cortisol, 17-ketosteroids, metanephrines and vanillylmandelic acid. As the patient was hypertensive, plasma aldosterone and plasma rennin were also tested. None of the tests indicated a functional adrenal tumor. The patient was under surveillance due to hepatitis B and had been subjected to AFP measurement and US scan 2, 8 and 14 months prior to his admission (AFP: 65, 69 and 60 ng/ml respectively). Regarding the facts that there was not remarkable increase in the AFP level during the last 14 months, the multiple US scans were negative and the CT scan on admission did not reveal any intrahepatic lesion, HCC was not considered in our patient.

**Figure 2:**
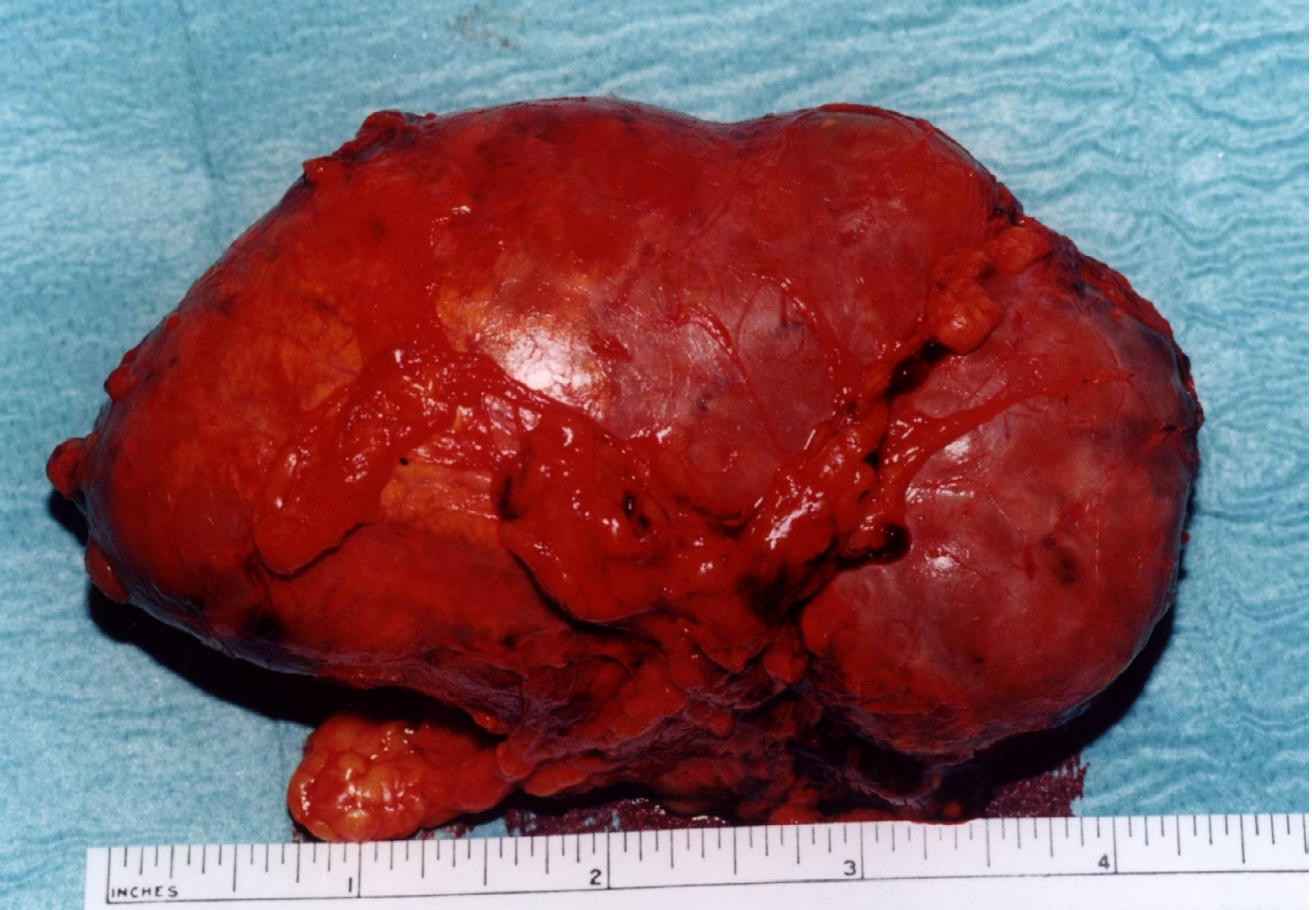
The resected adrenal mass was 11.5 cm by 7.5 cm in size.

With a presumptive diagnosis of a non-functional adrenal tumor, the patient underwent left adrenalectomy through a left Köcher incision. The exploration of the other intra-peritoneal organs did not reveal any other palpable lesions while the liver was found to be cirrhotic during the operation. The resected lesion was a firm elastic mass measuring 11.5 cm by 7.5 cm (Figure 2). The postoperative course was uneventful and the patient was discharged on day 7. Histologically, the resected lesion was a poorly differentiated metastatic HCC with trabecullar patterns. The patient was then informed of the findings and contacted for readmission, further investigation and adjuvant therapy. However, despite our repeated attempts, the patient refused to be subjected to any further investigation or treatment.

**Figure 3:**
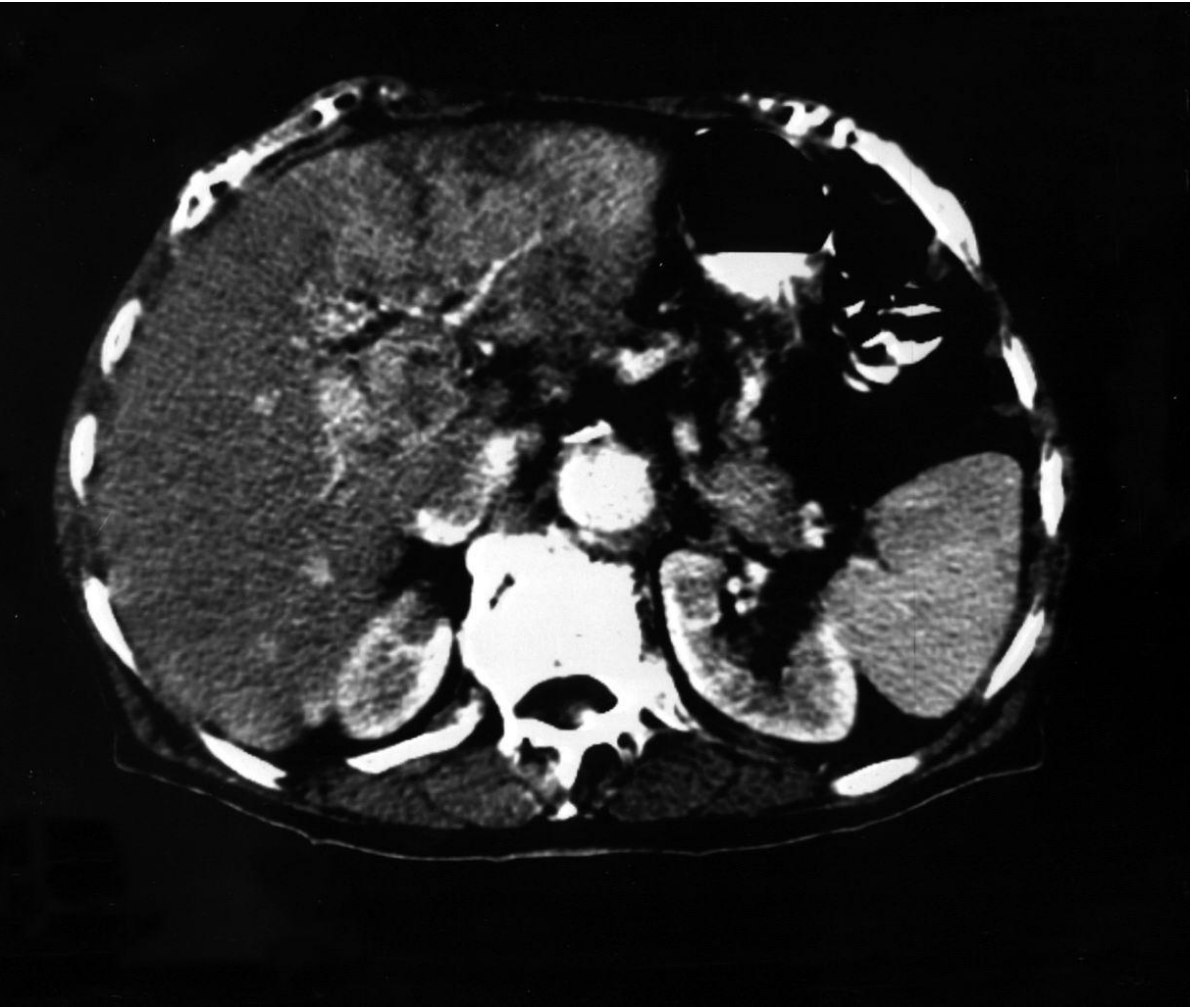
Hyperdense lesions of the liver in the arterial phase of the CT scan.

Seven months later patient presented with cachexia, icterus, abdominal discomfort and significant weight loss (more than 10 % of body weight). The serum alpha-fetoprotein (AFP) concentration was 1620.5 ng/ml and the serum bilirubin 5.4 mg/dL. CT scan revealed hyperdense lesions of the liver (Figure 3). Even though Trans-Arterial Embolization (TAE) was then performed, the serum AFP concentration showed persistently elevation to 3543 ng/ml one month later, and the patient died 3 months after readmission due to rapidly progressive liver failure.

## Discussion

Adrenal gland is the second most common site of hematogenous spread from HCC after the lung and has been found in up to 8.4% of cases at autopsy [1]. It is not clear why the adrenal gland has a greater incidence of metastases from HCC than other organs, however, it is claimed that the most likely route of metastases is the arterial spread via the aorta [3].

Adrenal metastasis in HCC are seldom treated by surgery as by the time they are diagnosed, the tumor is usually far advanced and the patients present with locally advanced primary hepatic lesions and poor liver function [4]. In our case, the adrenal metastasis was the first clinical presentation of HCC and till that time the primary tumor was not apparent on CT or US scan thus making it possible for us to resect the adrenal tumor.

At present, there is a wide variation in therapeutic modalities for adrenal metastases from HCC, as the tumor is usually accompanied by other distant or intrahepatic metastases and the patients are not always fit for a major operation. Trans-arterial embolization has been used for non-surgical treatment of these tumors. Sakamoto *et al *[5] stated that a complete TAE is difficult because the adrenal gland has three feeding arteries. Taniai *et al *[6] reported that despite the fact that adrenalectomy is the most effective way to control adrenal metastases from HCC, TAE is chosen as treatment of choice when the patients' performance status is too poor to tolerate an operation. Finally, ultrasound-guided percutaneous ethanol injection therapy (PEIT) is claimed to have technical difficulties and carry the additional risk of tumor seeding through the needle tract or bleeding from the punctured tumor [5,7]. However, Momoi *et al *[8] in a retrospective study of 20 patients treated for adrenal metastasis of HCC by various methods reported that PEIT shows good results for small adrenal metastasis. In our case, adrenalectomy was our first option as the patient's condition allowed an operation and the diagnosis of a primary HCC had not been established by the CT scan imaging, the serum AFP concentration and the liver function tests during the preoperative period.

Recent advances in diagnostic imaging techniques such as CT scan or Magnetic Resonance Imaging (MRI) have significantly improved the ability of detection of liver lesions while there have not been any reports of metastatic lesions in the adrenal gland without clinical or CT scan finding of the primary HCC [6]. Recently, Iannaccone *et al *[9] reported that despite the use of updated equipment and optimized study protocols, CT scan has poor sensitivity for the detection of hepatocellular carcinoma. This is in contrast to Bartolozzi *et al *[10] who reported that Lipiodol-CT scan is the single most sensitive examination to detect small nodules of HCC, having a specificity of 86% compared to 62% for conventional CT scan. Similarly, Kitagawa *et al *[4] stated that MRI is more useful than CT scan for the assessment of primary liver tumors. In our case, patient preference not to be subjected to further investigation immediately after the histological diagnosis of metastatic HCC, prevented further imaging of the liver with either MRI and/or Lipiodol-CT scan, in order to identify and locate the primary lesion which may have been resectable at this stage. Moreover, our patient had an abdominal ultrasound scan which did not reveal any intrahepatic lesion. The combination of US and CT scan appears to have a sensitivity of 86% in detecting hepatocellular carcinoma [10]. We assume that the intrahepatic lesion might have been a small one at that stage making difficult its detection by conventional imaging.

The review of the literature revealed a few reports of metastases being the initial presentation of HCC. Ascani *et al *[11] reported a case of an osteolytic vertebral lesion to be the first manifestation, which was clinically silent until then. Similar reports with an initial finding of skeletal metastases from clinically silent HCC have been also presented by Omura *et al *[12] and Yang *et al *[13] who reported two cases. Besides, Baba *et al *[14] reported histological confirmation of HCC with right atrial metastasis to be the primary clinical manifestation. Finally, Gilsanz *et al *[15] reported a case of a patient who had a mass in the gluteal region for three years that turned out to be a metastasis of HCC. To our knowledge, this is the first case with adrenal metastasis as first presentation and subsequent histological confirmation of a silent HCC.

Incidentally discovered adrenal masses (incidentalomas) are fairly common, but the correct management of such lesions is not well established. Every patient with an incidentally discovered adrenal mass has to be investigated to detect malignancy and subtle hormonal overproduction in order to select the cases for surgical treatment. There are reports mentioning the value of fine needle aspiration/cut biopsy in differentiation of benign and malignant incidentalomas, stating that they are more sensitive than CT and MRI scan while size criteria are of little value [16]. Other authors recommend the use of FNA cytology in all patients with incidentalomas of more than 2 cm in size [17]. However, it is well documented that regardless of the endocrine status of an adrenal mass, the mass size especially if it is greater than 6 cm is a parameter associated with malignancy [18,19]. Lesions greater than 6 cm in diameter have an approximate risk of 35% for malignancy [20]. Thus, many authors suggest that for prophylactic purposes, surgery should be considered in all patients with tumors greater than 6 cm [21-23]. This was also our policy as the adrenal mass in our patient was non functional and its size was greater than 6 cm suggesting high-risk of malignancy. However, if fine needle aspiration/tru-cut^® ^biopsy was performed, the primary tumor, which was obscure until then, might not have been missed until after the operation when the pathology report was available.

## Conclusion

Atypical presentations of HCC are rare, adrenal metastasis can be the first clinical manifestation of the disease. Fine needle aspiration/needle biopsy might be useful in the investigation of an accidentally discovered adrenal mass regardless of the size as it can lead to the detection of a primary tumor, in case of metastatic disease, that is silent at this stage.

## Competing interests

The author(s) declare that they have no competing interests.

## Authors' contributions

**KT**- conceived the study, performed the operation and coordinated the preparation of the manuscript for submission.

**EMZ**- did the literature search and drafted the manuscript.

**NS**- participated in the design of the study and preparation of manuscript for publication.

**IL**- participated in the design of the study and preparation of manuscript for publication.

**EVZ**- participated in the literature search and preparation of manuscript for publication and helped to draft the manuscript.

**DB**- has given final approval of the version to be published.
